# Radiomics Results for Adrenal Mass Characterization Are Stable and Reproducible Under Different Software

**DOI:** 10.3390/life15040560

**Published:** 2025-03-31

**Authors:** Giacomo Feliciani, Francesca Mascolo, Alberto Cossu, Luca Urso, Francesco Feletti, Enrico Menghi, Anna Sarnelli, Maria Rosaria Ambrosio, Melchiore Giganti, Aldo Carnevale

**Affiliations:** 1 Medical Physics Unit, IRCCS Istituto Romagnolo per lo Studio dei Tumori (IRST) “Dino Amadori”, 47014 Meldola, Italy; giacomo.feliciani@irst.emr.it (G.F.); enrico.menghi@irst.emr.it (E.M.); anna.sarnelli@irst.emr.it (A.S.); 2 Department of Translational Medicine—Section of Radiology, University of Ferrara, 44121 Ferrara, Italy; francesca.mascolo@edu.unife.it (F.M.); francesco.feletti@unife.it (F.F.); melchiore.giganti@unife.it (M.G.); 3 Department of Radiology and Laboratory Medicine, Arcispedale Sant’Anna University Hospital, 44124 Ferrara, Italy; a.cossu@ospfe.it; 4 Department of Translational Medicine—Section of Nuclear Medicine, University of Ferrara, 44121 Ferrara, Italy; luca.urso@unife.it; 5 Department of Medical Sciences—Section of Endocrinology, University of Ferrara, 44121 Ferrara, Italy; mariarosaria.ambrosio@unife.it

**Keywords:** radiomics, artificial intelligence, adrenal glands, computed tomography, adrenal incidentaloma

## Abstract

*Background*: This study aims to investigate stability and reproducibility of radiomics biomarkers for adrenal lesion characterization across different software packages. *Methods*: Unenhanced CT images from patients with adrenal tumors were analyzed. Radiomic features were extracted using SOPHIA Radiomics and SIBEX software. The datasets underwent Z-score normalization. Statistical comparisons were made using two-sample *t*-tests and Spearman correlation coefficients. Three classification models—Logistic Regression, Linear Discriminant Analysis, and Linear Support Vector Machine—were trained on the datasets. Model performance was evaluated using accuracy, precision, recall, F1 score, and ROC curves. Feature importance and the statistical significance of model performance differences were also analyzed. *Results*: The *t*-test results showed no significant differences in the radiomic features extracted by SOPHIA and SIBEX (*p*-values all equal to 1.0). Spearman correlation coefficients were high for most features, suggesting a strong similarity between the two software tools. Classification models generally performed better on the SOPHIA dataset, with higher accuracy and precision. Feature importance analysis identified “Quadratic mean” and “Strength” as consistently influential features. Paired *t*-tests indicated significant differences in accuracy and precision, while Wilcoxon signed-rank tests did not find significant differences across all performance metrics. *Conclusions*: Radiomic features extracted by SOPHIA and SIBEX are comparable, but slight variations in model performance highlight the need for standardized extraction protocols and fine-tuning of predictive features. The study underscores the importance of ensuring the stability and reproducibility of radiomics features for reliable clinical application in adrenal lesion characterization.

## 1. Introduction

The digitalization of clinical routine information has been a consistent trend in the field of medicine over the past few decades. New and increasingly sophisticated software was created to analyze the increasing amount of medical data that was available in digital format. Radiomics involves the extraction of a large number of quantitative features from medical images, which can then be used to develop predictive models for various clinical applications [1,2,3]. As artificial intelligence continues to advance at an unprecedented pace, radiomics has been extensively tested and applied in a variety of oncology-related areas, such as diagnosis, classification, prognostication, and treatment response assessment [2,4,5,6,7].

In recent years, an increasing number of studies have shown that radiomics could provide a risk-free and efficient method for enhancing the value of diagnostic imaging of adrenal masses with the aim of assisting in clinical decision-making [5,8,9].

However, a major challenge in radiomics methodology is the limited reproducibility of radiomic features under different experimental conditions and methodological approaches [10].

There is an unmet need to establish robust pre- or post-image acquisition methods for radiomics data harmonization: one of the key challenges in radiomics research is ensuring the stability and reproducibility of the extracted features and the derived biomarkers. Although radiomics provides a relatively objective and quantitative diagnostic pattern, several factors, such as image acquisition protocols, reconstruction parameters, and the choice of segmentation algorithms, can all introduce variability in every step of the radiomics workflow and could create an intrinsic methodological weakness in the whole process [5,11].

Zwanenburg et al. [12], through the work of the Image Biomarker Standardization Initiative (IBSI), provided a crucial framework for standardizing radiomic feature definitions to improve reproducibility across platforms. Despite this effort, practical implementations have shown that variability persists when features are extracted using different software packages.

To address these issues, researchers have explored various strategies to minimize the impact of such sources of variation, such as image resampling and batch effect correction [11,13]. Traverso et al. [14] conducted a comprehensive review of radiomics repeatability and reproducibility studies, emphasizing that inter-software differences and pre-processing steps remain critical sources of variation

In this study, we aim to further investigate the stability and reproducibility of radiomics biomarkers for adrenal lesion characterization. We will analyze the variability of radiomics features extracted from a dataset of unenhanced Computed Tomography (CT) images of adrenal tumors using various software packages, including both open-source and proprietary tools, and evaluate the performance of radiomics-based models built on these features.

To assess the stability and reproducibility of the radiomics biomarkers, we will evaluate the following aspects: consistency of radiomic feature values across different software tools; robustness of radiomics-based predictive models to changes in the feature extraction process.

The results of this study will contribute to a better understanding of the factors that influence the reproducibility of radiomics in the context of adrenal lesion characterization, possibly improving reproducibility and reliability of radiomics features and models.

## 2. Materials and Methods

### 2.1. Data Collection and Preprocessing

The datasets used in this study were obtained from unenhanced CT images of patients diagnosed with adrenal tumors who underwent adrenalectomy in our institution. Patient selection and lesion characteristics are consistent with those analyzed in our previously published study [8], and detailed data on patients and lesions are provided in the Supplementary Materials.

After manual segmentation, radiomic features were extracted using two different software packages: SOPHIA Radiomics (v 4-4.6.2; SOPHiA GENETICS SA. La Pièce 12, CH-1180. Rolle, Switzerland) and SIBEX (S-IBEX v 1.0). Each dataset included multiple radiomic features and histopathological labels indicating the diagnosis: 0 for adenoma and 1 for non-adenoma tumors.

To prepare the data for analysis, we performed Z-score normalization on all features except the outcome variable. Z-score normalization transforms the data to have a mean of 0 and a standard deviation of 1, which helps in comparing features that are on different scales. This preprocessing step ensures that the differences in the scale of measurements do not affect the comparative analysis between the two datasets.

### 2.2. Statistical Comparison and Correlation Analysis

We conducted a two-sample *t*-test for each feature to compare the distributions of the features extracted by SOPHIA and SIBEX. The *t*-test assesses whether the means of two independent samples are significantly different. A *p*-value less than 0.05 would indicate a statistically significant difference.

Additionally, we computed the Spearman correlation coefficients for each feature pair across the two datasets. Spearman correlation measures the strength and direction of association between two ranked variables. High correlation coefficients indicate that the features extracted by both software packages are similar.

### 2.3. Visualization

To visualize the distribution of features, we created boxplots for each feature in the two datasets. Boxplots provide a graphical summary of the central tendency, variability, and distribution shape of a dataset. We included the *t*-test *p*-values in the boxplot titles to highlight the statistical significance of the differences between the datasets.

### 2.4. Model Training and Evaluation

We trained three different classification models on each dataset separately:

1. Logistic Regression: A linear model used for binary classification.
2. Linear Discriminant Analysis (LDA): A linear classifier that assumes normal distribution of the data and equal covariance matrices for each class.
3. Linear Support Vector Machine (SVM): A linear classifier that finds the hyperplane that best separates the data into classes.

For each model, we evaluated the following performance metrics:

1. Accuracy: The proportion of correctly classified instances.
2. Precision: The proportion of true positive instances among the instances classified as positive.
3. Recall: The proportion of true positive instances among the actual positive instances.
4. F1 Score: The harmonic mean of precision and recall.

We also plotted ROC curves to visualize the performance of each model. The ROC curve is a graphical representation of the true positive rate versus the false positive rate at various threshold settings. The area under the ROC curve (AUC) provides a single measure of overall model performance.

### 2.5. Feature Importances

For each model, we extracted and visualized the feature importances. Feature importance provides insight into which features contribute most to the model’s predictions. For linear models like Logistic Regression and Linear SVM, feature importance is derived from the model coefficients. For LDA, feature importance is related to the linear discriminants.

### 2.6. Statistical Significance of Model Performance

To assess whether the differences in model performance between the two datasets were statistically significant, we conducted paired *t*-tests and Wilcoxon signed-rank tests for each performance metric. The paired *t*-test compares the means of two related groups, while the Wilcoxon signed-rank test is a non-parametric test that compares the ranks of paired samples. The null hypothesis of the tests was that there is no statistically significant difference in the numerical values of the features extracted by the two software tools (SIBEX and SOPHIA).

## 3. Results

This study is based on a population consisting of 48 patients (26 males, 22 females) accounting for 50 lesions (24 in the female population, 26 in the male). The age of patients ranged between 27 and 86 years old, with an average age of 72 for women and 70 for men. In Supplementary Table S1, we provide specific information about the patients.

### 3.1. Statistical Comparison and Correlation Analysis

**Figure 1:**
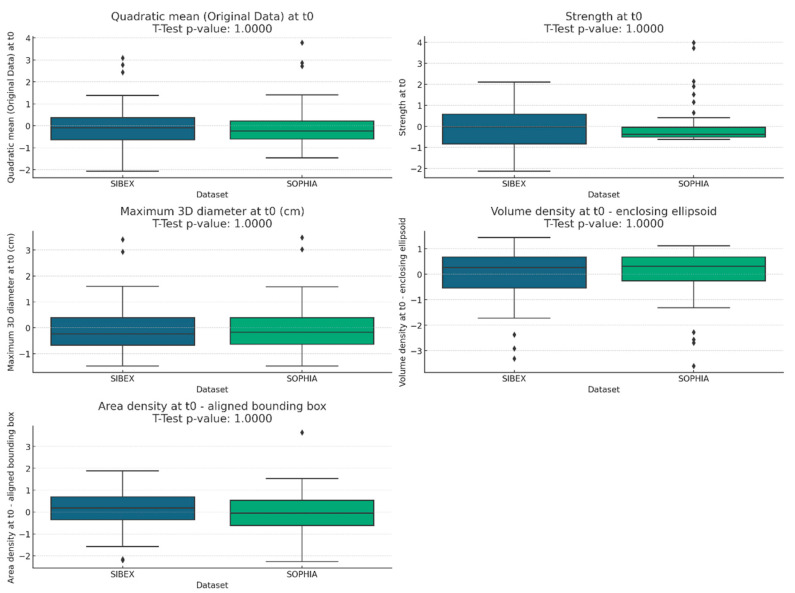
**Boxplots comparing radiomic feature distributions between SOPHIA and SIBEX datasets.** Boxplots illustrate the distribution of selected radiomic features extracted using SOPHIA and SIBEX software. The visual overlap between boxplots and the inclusion of *t*-test *p*-values in each plot title confirm the absence of significant differences in feature means.

The *t*-test results showed that there were no statistically significant differences between the features extracted by SOPHIA and SIBEX, with all *p*-values being 1.0. This suggests that the mean values of the features from the two datasets are very similar, as shown in Table 1 and Figure 1.

The Spearman correlation analysis revealed high correlation coefficients for most features, as shown in Table 2, indicating a strong relationship between the features extracted by the two software packages. The highest correlation was observed for “Maximum 3D diameter at t0 (cm)” with a coefficient of 0.967, suggesting almost identical measurements by both software packages for this feature. Strength, volume density, and area density show an unexpected mid-level correlation of 0.74, 0.48, and 0.51. This mid level correlation is unexpected, in particular, for volume density and area density, which are shape features that depend only on contouring of the lesion.

### 3.2. Visualization

The boxplots for each feature showed similar distributions between the SOPHIA and SIBEX datasets. The inclusion of *t*-test *p*-values in the titles confirmed the lack of statistically significant differences. This visual analysis supports the conclusion that the features extracted by both software packages are comparable, as shown in Figure 1.

### 3.3. Model Performance

The performance of the classification models on the SIBEX dataset was as follows:

1. Logistic Regression: Accuracy 0.72, Precision 0.730, Recall 0.871, F1 Score 0.794
2. Linear Discriminant Analysis: Accuracy 0.72, Precision 0.718, Recall 0.903, F1 Score 0.800
3. Linear SVM: Accuracy 0.66, Precision 0.719, Recall 0.742, F1 Score 0.730

On the SOPHIA dataset, the performance metrics were as follows:

1. Logistic Regression: Accuracy 0.90, Precision 0.882, Recall 0.968, F1 Score 0.923
2. Linear Discriminant Analysis: Accuracy 0.84, Precision 0.871, Recall 0.871, F1 Score 0.871
3. Linear SVM: Accuracy 0.90, Precision 0.882, Recall 0.968, F1 Score 0.923

These results indicate that the models generally performed better on the SOPHIA dataset compared to the SIBEX dataset. Logistic Regression and Linear SVM had notably higher accuracy, precision, recall, and F1 scores on the SOPHIA dataset (Table 3).

**Figure 2:**
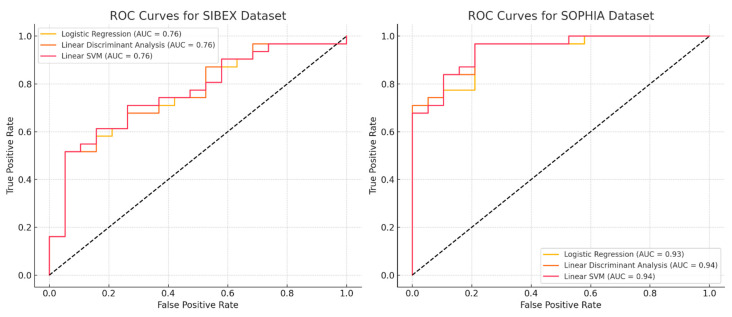
**ROC curves for classification models trained on SOPHIA and SIBEX datasets.** ROC (Receiver Operating Characteristic) curves depicting the performance of Logistic Regression, LDA, and Linear SVM models on SOPHIA and SIBEX datasets. The area under the curve (AUC) highlights superior predictive accuracy for models based on SOPHIA-extracted features, particularly for Logistic Regression and Linear SVM. Differences in AUC values further underscore the impact of feature extraction variability on model performance, highlighting the impact of software choice in radiomics workflows.

In Figure 2, we show the ROC curves of the three models for the two software tools.

### 3.4. Feature Importances

**Figure 3:**
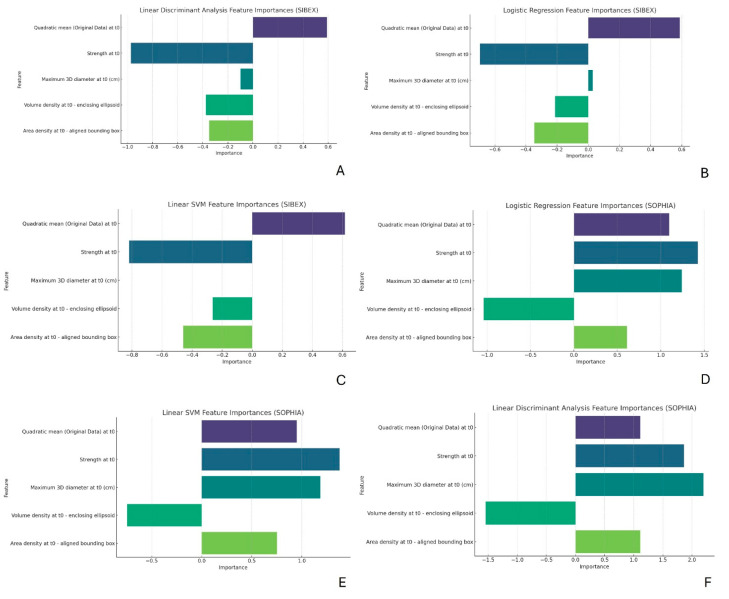
(**A**–**F**): **Feature importance analysis for predictive models.** Bar charts representing the importance of radiomic features in Logistic Regression, LDA, and Linear SVM models. “Quadratic mean” and “Strength” consistently emerge as key predictive features, despite minor variations in their ranked importance across datasets. Differences in “Volume density” and “Area density” values between SOPHIA and SIBEX datasets are evident, aligning with observed discrepancies in Spearman correlations and suggesting a need for fine-tuning before these features can be used reliably across different software and institutions.

The feature importance analysis shown in Figure 3A–F highlighted that “Quadratic mean (Original Data) at t0” and “Strength at t0” were among the most influential features across all models and datasets. Although the rankings and values of these importances varied slightly between models and datasets, their overall importance remained consistent. Statistical tests and correlation analyses revealed that the values of “Maximum 3D diameter” were similar between SOPHIA and SIBEX, as indicated by non-significant *p*-values. However, this feature was more important in the SOPHIA models, suggesting better integration or interaction with other features in the dataset. Conversely, the “Strength”, “Volume density”, and “Area density” features exhibited differences in values between the two software packages, even though these differences were not statistically significant. These discrepancies in feature importances likely contribute to the observed differences in model prediction performance. Considering the differences in Spearman correlation as well, it is evident that even though both software tools are IBSI compliant, these features require fine-tuning before the software can be used independently.

### 3.5. Statistical Significance of Model Performance

The paired *t*-test results indicated statistically significant differences in accuracy (*p*-value 0.035) and precision (*p*-value 0.001) between the models on the two datasets. However, the recall (*p*-value 0.324) and F1 score (*p*-value 0.065) differences were not statistically significant.

The Wilcoxon signed-rank test results showed no statistically significant differences in any of the performance metrics (accuracy, precision, recall, and F1 score) between the models on the SIBEX and SOPHIA datasets. The *p*-values for all metrics were above the common significance threshold of 0.05, suggesting that the observed performance differences might not be robust.

## 4. Discussion

Overall, our analysis indicates that the radiomic features extracted by SOPHIA and SIBEX are comparable but not directly usable and require fine-tuning. The models trained on the SOPHIA dataset generally exhibited better performance metrics, but the differences were not statistically significant according to the Wilcoxon signed-rank test. However, the two packages should not be used independently without proper fine-tuning of the features that are required by the model. These considerations are essential for acknowledging the role of radiomics in clinical practice and, in our specific case, for understanding its impact on the management of adrenal lesions.

Recent literature has increasingly demonstrated that radiomics may offer a safe and effective approach to improving the diagnostic value of imaging for adrenal masses, supporting clinical decision-making [3,5,8,9,15].

An adrenal incidentaloma refers to an adrenal mass that is discovered during imaging studies that were not specifically performed to investigate a suspected adrenal disease [16,17]. This is a commonly encountered scenario in clinical practice. In fact, the prevalence of adrenal incidentalomas has been steadily increasing due to the growing use of cross-sectional imaging. In many instances, these unexpected adrenal masses are benign.

Particularly when the initial imaging characteristics are nonspecific, these incidental findings pose diagnostic challenges for both radiologists and referring clinicians. While the majority of lesions are benign, mainly representing harmless non-functioning adenomas, a small number may be hormone-secreting, malignant, or both and cannot be accurately identified through imaging alone [17,18]. In this scenario, after a thorough clinical assessment, several strategies may be implemented: Additional imaging after a first inconclusive unenhanced CT, including MR or contrast-enhanced CT with a delayed washout analysis; follow-up imaging, which may be beneficial to illustrate the natural history; or adrenalectomy [19,20].

To better define therapeutic strategies, diagnostic imaging is crucial for categorizing adrenal lesions, as it can identify the precise etiology of various conditions using CT and magnetic resonance imaging (MRI), thereby eliminating the need for additional tests. On the other hand, patients with an indeterminate adrenal mass on imaging, notably unenhanced CT, will require further evaluation by a multidisciplinary team acknowledging the patient’s clinical context. The requirement for further examinations exposes patients to avoidable injury resulting from potentially unnecessary diagnostic or invasive procedures, hence contributing to an obvious increase in healthcare costs [21,22]. In this framework, radiomics can provide further insight into adrenal incidentaloma characterization through the analysis of baseline CT scans.

CT and MRI play crucial roles in abdominopelvic oncologic and non-oncologic imaging, and they are frequently employed for the screening, initial evaluation, staging, subtype definition, response assessment, and outcome prediction of abdominopelvic neoplasms [23,24,25]. The availability of open-source and commercial software packages for radiomics analysis, as well as recent advancements in high-performance computing capabilities, has enabled the processing of large volumes of images and the extraction of high-throughput quantitative features.

Recent studies have emphasized the importance of robust imaging techniques in clinical diagnostics, particularly in complex conditions, underscoring the need for advanced and reliable imaging biomarkers which are crucial for enhancing diagnostic accuracy and treatment outcomes, thus guiding effective clinical interventions. In this context, radiomics and other artificial intelligence tools may be useful in overcoming conventional image assessment constraints and increasing the utility of diagnostic imaging [26].

Nevertheless, the radiomics workflow is characterized by an intrinsic methodological shortcoming that has been acknowledged since the inception of radiomics analysis, which results from the numerous sources of variation present in each phase. This research frequently fails to adequately consider sources of variation and reports isolated results that are not validated by replication in external datasets, despite an explosive increase in the radiomics literature [11,26]. The tempo of innovation in radiomics is slowed, and its translational potential is restricted by the resulting concerns about rigor and reproducibility [27].

Indeed, radiomics is a rapidly evolving field, and the extraction of reliable and reproducible features is critical for its clinical application [12]. To enhance trust in radiomics results, future studies must address several crucial steps. First, it is essential to identify all potential factors that may influence feature reproducibility in a specific experimental setup. This process should be conducted systematically, with a dedicated analysis for each contributing factor. Significant efforts have already been made by the Image Biomarker Standardization Initiative (IBSI) to define parameters and convolutional filters that facilitate the extraction of reliable radiomics features [10,12].

It is important to note that, despite both SOPHIA Radiomics and SIBEX being IBSI-compliant, software-specific differences in feature extraction pipelines—including variations in interpolation methods, intensity discretization approaches, and segmentation handling—represent inherent limitations that may influence feature reproducibility and downstream model performance. These implementation-dependent effects have been extensively documented in the radiomics literature and remain a recognized challenge when comparing results across different software platforms, further supporting the need for tailored harmonization strategies in multicenter studies.

In particular, previous studies have demonstrated the importance of comparing radiomic features across different software tools to ensure consistency and reliability in clinical decision-making.

A study by Zwanenburg et al. [28] evaluated the variability of radiomic features extracted from various software tools and highlighted significant discrepancies that could impact clinical outcomes. Similarly, Traverso et al. [14] assessed the reproducibility of radiomic features in multi-center studies and found that feature extraction software significantly influenced the results, emphasizing the need for standardized extraction protocols.

In line with these findings, our study compares radiomic features extracted by SOPHIA Radiomics and SIBEX, focusing on adrenal tumors. Our results show no statistically significant differences in the mean values of the features extracted by the two software tools, suggesting that both can be reliably used for feature extraction. High Spearman correlation coefficients for most features further support the consistency of the features across the two software packages.

We evaluated the performance of three classification models—Logistic Regression, Linear Discriminant Analysis (LDA), and Linear Support Vector Machine (SVM)—on both datasets. The models generally performed better on the SOPHIA dataset, particularly in terms of accuracy and precision. This difference could be attributed to slight variations in feature extraction methodologies between the two software tools. The main difference between the models appears to lie in the importance of the shape features, which is somewhat unexpected, as they are commonly regarded in the literature as the most stable features. However, fine-tuning these features for further comparison is relatively straightforward and could potentially eliminate any discrepancies between the two software programs.

The feature importance analysis revealed that certain features, such as “Quadratic mean” and “Strength”, were consistently important across all models and datasets. This consistency aligns with the findings of Parmar et al. [29], who identified key radiomic features that are robust and predictive across different cancer types and imaging protocols. However, “Strength” exhibited slightly different values between the two software packages. Although these differences were not statistically significant, we suggest fine-tuning this feature.

The quadratic mean is a first-order feature derived from a histogram, showing a moderate correlation with the HU median (or mean) value, as demonstrated in studies by Zhang et al. [17], for distinguishing lipid-poor adenomas from other histotypes. In contrast, strength is a more complex second-order feature associated with image texture, particularly correlating with the concept of coarseness, as we described in our previous paper [8].

Furthermore, “Maximum 3D diameter” showed no differences in numerical values of feature extraction; however, its importance dropped to zero when modeling prediction performances across all models. This can be explained by poor interaction with other features using the SIBEX software, necessitating fine-tuning of the other features as described next. “Volume density” and “Area density” also showed different values, albeit without statistical significance, and unexpectedly lower correlations of 0.48 and 0.51. These differences can be attributed to the different masking methods used by the extraction software, even though the IBSI initiative reports these features as strong across different software extractions. Fine-tuning these features is strongly recommended before using the software independently across institutions.

We performed paired *t*-tests and Wilcoxon signed-rank tests to determine if the differences in model performance between the two datasets were statistically significant. While the *t*-tests indicated significant differences in accuracy and precision, the Wilcoxon tests did not find statistically significant differences in any performance metrics. These conflicting results can be explained by the differences in feature values and their interaction outlined before, requiring further fine-tuning for the independent use of the two software tools.

Future studies could explore advanced methodologies such as AutoML and ensemble learning to further optimize radiomics-based classification models. AutoML has been demonstrated to enhance feature selection and model optimization in medical imaging applications [30], while ensemble learning approaches have shown promise in improving classification robustness [31]. Incorporating these techniques could refine predictive performance and facilitate the generalizability of radiomic models across different datasets and clinical settings

Several limitations must be considered when interpreting our results. The sample size is relatively small, and the data are from a single institution, which may limit the generalizability of the findings. Additionally, only three linear models were used, and feature selection was not performed, potentially affecting the robustness of the results. Future studies should include larger, multi-center datasets, use more complex models, and integrate clinical data to provide a more comprehensive evaluation.

## 5. Conclusions

Our study contributes to the growing body of literature comparing radiomic features extracted by different software tools. The findings suggest that both SOPHIA Radiomics and SIBEX can be reliably used for extracting radiomic features from adrenal tumor CT images. However, slight variations in model performance highlight the need for standardized extraction protocols and the necessity to fine-tune predictive feature case by cases beyond IBSI initiative guidelines. Only through these considerations can we confidently integrate radiomics into clinical practice and, specifically in our case, fully appreciate its potential impact on the management of adrenal lesions.

## Figures and Tables

**Table 1:** ***t*-test results for radiomic features extracted using SOPHIA Radiomics and SIBEX software**. Comparison of mean values for radiomic features, as extracted by the two software tools. The t-statistic and corresponding *p*-values (all at 1.0) confirm the absence of statistically significant differences between the datasets.

Radiomic Feature	t_stat	*p*_value
Quadratic mean	−0.84	1.00
Strength	0.97	1.00
Maximum 3D diameter	2.20	1.00
Volume density—enclosing ellipsoid	−0.81	1.00
Area density—aligned bounding box	−0.39	1.00

**Table 2:** **Spearman correlation coefficients for radiomic features across software**. Correlation analysis between features extracted by SOPHIA and SIBEX software tools. High coefficients (e.g., 0.81 for “Quadratic mean” and 0.97 for “Maximum 3D diameter”) indicate strong concordance, while moderate correlations (e.g., 0.48 for “Volume density”) suggest variability potentially linked to differences in contouring algorithms. These insights highlight critical areas for further harmonization efforts in feature extraction protocols.

Radiomic Feature	Spearman Correlation	*p*-Value
Quadratic mean	0.81	<0.01
Strength	0.74	<0.01
Maximum 3D diameter	0.97	<0.01
Volume density—enclosing ellipsoid	0.48	<0.01
Area density—aligned bounding box	0.51	<0.01

**Table 3:** **Performance metrics of classification models trained on SOPHIA and SIBEX datasets**. The table summarizes the accuracy, precision, recall, and F1-score for each model. Overall, models trained on the SOPHIA dataset consistently outperformed those trained on SIBEX data, especially for Logistic Regression and Linear SVM. Although not always statistically significant, these differences point to the potential impact of software-specific extraction methodologies on predictive performance.

SIBEX software	Accuracy	Precision	Recall	F1 Score
Logistic Regression	0.72	0.73	0.87	0.79
Linear Discriminant Analysis	0.72	0.72	0.90	0.80
Linear SVM	0.66	0.72	0.74	0.73
**SOPHIA software**	**Accuracy**	**Precision**	**Recall**	**F1 Score**
Logistic Regression	0.90	0.88	0.97	0.92
Linear Discriminant Analysis	0.84	0.87	0.87	0.87
Linear SVM	0.90	0.88	0.97	0.92

## Data Availability

The data presented in this study are available on request from the corresponding author due to ethical reasons.

## Supplementary Materials

The following supporting information can be downloaded at: https://www.mdpi.com/article/10.3390/life15040560/s1, Table S1: Features of the patients and lesions analyzed in the study.

## Abbreviations

The following abbreviations are used in this manuscript:

AUC	Area Under the Curve
CT	Computed Tomography
IBSI	Image Biomarker Standardization Initiative
LDA	Linear Discriminant Analysis
MRI	Magnetic Resonance Imaging
SVM	Support Vector Machine
