# Supplementary material for: Radiomics Results for Adrenal Mass Characterization Are Stable and Reproducible Under Different Software
Source: Life (Basel). 2025 Mar 31;15(4):560. doi: 10.3390/life15040560 (PMC12028440; doi:10.3390/life15040560)
Supplement: Supplementary file 1 [file life-15-00560-s001.zip › life-3492004-supplementary.pdf]

Supplementary materials

Characteristics of patients and adrenal tumors

This retrospective study was conducted in a single institution.

From an initial dataset of 251 patients who underwent adrenalectomy at our institution, the final study population comprised 48 patients with 50 adrenal lesions, after applying inclusion and exclusion criteria.

Inclusion criteria included the following: solid focal adrenal lesions treated with adrenalectomy at our institution, availability of a complete histopathology report, pre-operative non-enhanced computed tomography (CT) scan, and lesions with calcifications, gross fat components, or a median attenuation below 10 Hounsfield Units (HU).

Exclusion criteria included lesions with a maximum diameter smaller than 1 cm and infiltrative lesions.

The characteristics of the patients and lesions included in the final analysis are reported in Table S1.

Patients (n)	48
males (n)	22
females (n)	26
Age (average; range, years)	61; 27–86
Lesions (n)	50
bilateral (n; %)	4
Diameter (average; range, cm)	5.5; 1.5–14.7
Histology (n; %)	
adenoma	19; 38%
pheocromocytoma	9; 18%
metastasis	8; 16%
adrenal carcinomas	5; 10%
myelolipoma	7; 14%
other histology	2; 4%

Table S1. Features of the patients and lesions analyzed in the study.

CT data, imaging analysis, and segmentation

Each CT scan, retrieved by querying the institutional Radiological Information Systems - Picture Archiving and Communication system (RIS-PACS), was independently reviewed by two abdominal radiologists with 3 and 10 years of experience, respectively. Lesions with calcifications, gross fat component, or a median attenuation of less than 10 HU, were excluded. A single region of interest (ROI) was placed on the axial plane, covering more than 50% of the target lesion. Discrepancies between the two readers were resolved by consensus.

The CT studies that met inclusion and exclusion criteria were acquired on four different multidetector scanners: Philips Brilliance 64, GE Lightspeed VCT, Philips iCT 256, and Siemens Biograph 64. The acquisitions followed standard protocols adjusted for patient biometrics (10–400 effective mAs, 120 kVp, 1.375–1.75 pitch, and 1.5–3 mm slice thickness). Images were reconstructed using standard soft-tissue kernels, specific to each scanner model.

A final-year radiology resident retrieved the fully anonymized CT images from the RIS-PACS database in DICOM format. Manual segmentation of adrenal lesions was performed using 3DSlicer software with the SlicerRT extension, creating a three-dimensional contoured volume of interest (VOI). The VOI encompassed the entire adrenal mass, avoiding adjacent peri-adrenal tissues such as fat, vessels, and neighboring organ parenchyma. The appropriateness of the contours was verified by the same two experienced abdominal radiologists. The segmented VOIs were exported as DICOM files with RT options enabled.

Following segmentation, the images were processed using SOPHiA DDMTM Radiomics software (compliant with the Image Biomarker Standardization Initiative, IBSI). Images were resampled to 1/1/1 mm resolution for standardization, with grey-level quantization performed at 32 bins. Radiomics analysis extracted 209 imaging features from each segmented volume, including first-, second-, and higher-order features. First-order features were derived from voxel intensity histograms, while second- and higher-order features were calculated using intensity size-zone, co-occurrence, and run-length-based matrices. The 209 features extracted are fully described in the IBSI Reference Handbook.
